# CDK7 inhibition suppresses rheumatoid arthritis inflammation *via* blockage of NF‐κB activation and IL‐1β/IL‐6 secretion

**DOI:** 10.1111/jcmm.13414

**Published:** 2017-10-30

**Authors:** Honghai Hong, Yingmin Zeng, Wenxuan Jian, Lei Li, Liying Lin, Yousheng Mo, Meiling Liu, Shuhuan Fang, Yong Xia

**Affiliations:** ^1^ Department of Clinical Laboratory Third Affiliated Hospital of Guangzhou Medical University Guangzhou China; ^2^ DME center Clinical Pharmacology Institute Guangzhou University of Chinese Medicine Guangzhou China; ^3^ Department of Reproductive Medicine Center Key Laboratory for Reproductive Medicine of Guangdong Province Third Affiliated Hospital of Guangzhou Medical University Guangzhou China

**Keywords:** rheumatoid arthritis, CDK7, BS‐181, NF‐κB signalling

## Abstract

Rheumatoid arthritis (RA) is a chronic inflammatory disease characterized by joint swelling, joint tenderness and destruction of synovial joints, leading to severe disability. Anti‐inflammatory drugs and disease‐modifying anti‐rheumatic drugs (DMARDs) may improve RA process. However, in most patients the treatment effect is still not satisfactory. Cyclin‐dependent kinase 7 (CDK7) plays a well‐established role in the regulation of the eukaryotic cell division cycle, and recent studies indicated that it exerted anti‐inflammatory effect. In our previous research, we found that inhibition of CDK7 by highly selective inhibitor BS‐181 significantly impeded the development of collagen‐induced arthritis (CIA) mice. However, the underlying mechanism of CDK7 in RA remains to be explored. We elucidated the molecular mechanism of CDK7 inhibition in RA inflammation by administration of CDK7 highly selective inhibitor BS‐181 and siRNA‐CDK7. We found that both IL‐1β, IL‐6, IL‐8 and RANKL transcript levels and IL‐1β/IL‐6 secretion were effectively suppressed by BS‐181 treatment as well as CDK7 knockdown. Furthermore, CDK7 inhibition prevented NF‐κB signalling pathway activation and restrained p65 nuclear translocation. Moreover, CDK7 selective inhibitor BS‐181 also blocked phosphorylation of p65 in MH7A cells. These results strongly indicate that CDK7 inhibition by BS‐181 and siRNA‐CDK7 significantly suppresses rheumatoid arthritis inflammation, which may be *via* blockage of NF‐κB signalling pathway and IL‐1β/IL‐6 secretion.

## Introduction

Rheumatoid arthritis (RA) is a chronic inflammatory disease characterized by joint swelling, joint tenderness and destruction of synovial joints, leading to severe disability 1, 2. It is more common in females and affects around 0.5–1.0% of adults in the developed world 3. Biological therapies that target a specific inflammatory pathway or immune‐related system could improve outcomes of RA patients, contributing to reduced mortality and comorbidity 4. Currently, there is no preventive treatment or cure for RA. The primary treatment is usually disease‐modifying anti‐rheumatic drugs (DMARDs), in particular the anchor DMARD methotrexate (MTX) 5, 6,which reduce synovitis and systemic inflammation. Biological agents, such as antibodies that block tumour necrosis factor (TNF), have been used to treat RA patients who have failed to respond to treatment with conventional DMARDs 7, 8. Nevertheless, around one‐third of anti‐TNF‐treated patients do not respond 9, 10. Therefore, further studies are required to meet the pressing clinical need for novel therapies.

The cyclin‐dependent kinases (CDKs) are serine–threonine kinases that tightly regulate progression through the G1, S, G2 and M phases of the cell cycle 11. Activation of specific CDKs is required for the appropriate progression through the cell cycle and into the next stage of the cell cycle. The control of gene transcription involves a set of cyclin‐dependent kinases (CDKs), including CDK7, CDK8, CDK9, CDK12 and CDK13, that play essential roles in transcription initiation and elongation by phosphorylating RNA polymerase II (RNAPII) and other components of the transcription apparatus 12, 13, 14. Recent studies show that CDKs also play a key role in inflammatory response 15. Inhibition of CDK4 and CDK6 activity suppresses MMP‐3 and IL‐1R secretion in synovial cells, impeding inflammatory reaction of rheumatoid arthritis through dependent or non‐dependent Rb 16, 17, 18. CDK5 promotes the differentiation of monocytes and regulates neutrophil secretory function *via* binding to p53 19, 20. It has been discovered that CDK7 inhibition could promote resolution of inflammation in bleomycin‐induced lung injury model 21. In our previous research, we found that CDK7 highly selective inhibitor BS‐181 significantly impeded the development of collagen‐induced arthritis (CIA) mice 22. However, the underlying mechanism of CDK7 in RA remains to be explored. Here, we elucidated the molecular mechanism of CDK7 inhibition in RA by siRNA‐CDK7 and administration of CDK7 highly selective inhibitor BS‐181, a pyrazolo[1,5‐α] pyrimidine‐derived compound 23.

## Materials and methods

### Cell culture

Human rheumatoid fibroblast‐like synoviocyte MH7A cells used in this study were purchased from Guangzhou Jinyu company Guangzhou, Guangdong Province, China. MH7A cells were cultured in DMEM medium supplemented with 15% foetal bovine serum (100 U/ml penicillin and 100 g/ml streptomycin (Gibco, Carlsbad, CA, USA)) at 37°C in a humidified atmosphere of 5% CO^2^ in air. The protocol for collecting fibroblast‐like synovial (FLS) cells from CIA mice's synovium was referred to Kono *et al*. 17. Briefly, the synovium was removed, minced and placed in 10 ml Hanks’ balanced salt solution containing type‐I collagenase (Sigma‐Aldrich, San Francisco, USA). After a 2‐hrs digestion at 37°C, each digest was sequentially passed through a metal mesh and then a nylon mesh with 100 μm pores. The liberated cells were collected by centrifugation and placed in a 75‐cm^2^ culture flask containing 15 ml of Iscove's modified Dulbecco's medium (IMDM, Sigma‐Aldrich) supplemented with 10% heat‐inactivated FBS, 100 IU/ml penicillin and 100 μg/ml streptomycin. The liberated cells were then cultured at 37°C in a humidified atmosphere of 5% CO2. Passage 3‐6 FLS cells were subjected to the experimental procedures noted below.

### Quantitative real‐time PCR

The MH7A and FLS cells were stimulated by 1 μg/ml LPS and treated with 80 nM BS‐181 for 24 hrs. Total RNA was extracted from MH7A and FLS cells according to the manufacturer's instructions for TRIzol reagent (Invitrogen, CA, USA). Total RNA (500 ng) was used for reverse transcription using PrimeScript RT reagent Kit Perfect Real‐Time kit (Takara Bio Inc., Shiga 525‐0058, Japan). The cDNA was used for quantitative real‐time PCR analysis (qPCR) using SYBR Premix Ex Taq™ (Takara Bio Inc.) and an Roche's capillary‐based Light Cycler 2.0 Systems (Roche Diagnostics Corporation, Indianapolis, IN, USA). Cells cDNA was amplified with specific primers for IL‐1β (sense primer: AACAGGCTGCTCTGGGATTC, antisense primer: AGTCATCCTCATTGCCACTGT), IL‐6 (sense primer: AGTTCCTGCAGAAAAAGGCAAAG, antisense primer: AAAGCTGCGCAGAATGAGATG), IL‐8 (sense primer: ACCGGAAGGAACCATCTCAC, antisense primer: TGGCAAAACTGCACCTTCACAC), RANKL (sense primer: GGAGTTGGCCGCAGACAAGA, antisense primer: ATTAGGATCCATCTGCGCTCTG) and β‐actin (sense primer: ACTCTTCCAGCCTTCCTTC, antisense primer: ATCTCCTTCTGCATCCTGTC) (Invitrogen). Target mRNA was determined using the comparative cycle threshold method of relative quantitation. β‐actin was used as an internal control.

### Measurements of supernatant IL‐1β and IL‐6

We collected the supernatant of MH7A and FLS cells, which were stimulated by 1 μg/ml LPS and treated with 80 nM BS‐181 for 48 hrs. The levels of IL‐1β and IL‐6 were measured by ELISA (R&D Systems, Minneapolis, USA) according to the manufacturer's instructions. Optical density (OD) values were measured at 450 nm.

### Cell transfection and siRNA for CDK7

The protocol for transfection was referred to Fan *et al*. 13.. Transfection was conducted with the Lipofectamine 2000 (Invitrogen) according to the manufacturer's instructions. SiRNA was synthesized by Ribobio (siCDK7‐1: 5′ CCGCCUUAAGAGAGAUAAA dTdT 3′ and siCDK7‐2: 5′ CAACAUUGGAUCCUACAUA dTdT 3′), and the end concentration of siRNA was 20 μM. MH7A cells were transfected in six‐well plates with a total of 20 μl siRNA and 8 μl Lipo2000 in a serum‐free culture medium. After 4–6 hrs, the medium was replaced by complete medium with 10% FBS and antibiotics.

Twenty‐four hours later, the cells were harvested for the analysis of IL‐1β, IL‐6, IL‐8 and RANKL expression.

### Immunofluorescent assay

LPS and BS‐181 were added into the MH7A cells which were grown on a glass coverslip placed in a six‐well plate for 24 hrs. MH7A cells were fixed with 4% paraformaldehyde for 15 min. The cells were blocked with goat serum for 30 min. Then, the cells were incubated with primary antibodies against p65 (1:100 dilution) (Santa Cruz, Texas 75220, USA) overnight at 4°C and incubated with fluorescence‐conjugated secondary antibody (Alexa Fluor 488 or 594 antibodies, 1:2000 dilution) (Life Technologies, Massachusetts, USA) at 37°C for an hour. DAPI (1:5000) were for staining cell nuclear.

### Western blot

LPS‐induced MH7A cells treated with BS‐181 for 24 hrs were harvested and lysed after triple PBS washings. Protein concentration was determined using Bio‐Rad DC protein assay kit (Bio‐Rad Laboratories, Hercules, CA, USA) according to the manufacturer's protocol. Aliquots of equal amounts of protein (80 μg) from the lysate underwent Western blot analysis for the IkB kinase (IKK)‐β (CST 8943S, diluted 1:2000), p‐IKK‐β (CST 8943S, diluted 1:2000), p‐IKB‐α (CST 2859S, diluted 1:2000), IKB‐α (CST 11930S, diluted 1:2000), p65 (Santa Cruz sc‐71675, diluted 1:1000) and p‐p65 (Santa Cruz sc‐135768, diluted 1:1000), histone 3 (Abcam, San Francisco, USA ab1791, diluted 1:2000) and β‐actin (Sigma‐Aldrich A1978, diluted 1:10000). The primary antibodies were incubated overnight at 4°C, followed by washing with TBST and incubating with peroxidase‐labelled secondary antibody (from Vector). Protein visualization was achieved using enhanced chemiluminescence detection reagents.

### Nuclear and cytoplasmic protein extraction

Nuclear and cytoplasmic protein extraction was carried out according to Thermo NE‐PER Nuclear and Cytoplasmic Extraction Reagents kit manufacturer's instructions. Briefly, 1 × 10^7^ MH7A cells treated with LPS and BS‐181 for 48 hrs were harvested with 200 μl CER I buffer, intense vortex shock 15 sec., the cell is fully suspended. Incubation on ice for 10 min. and addition of CER II buffer, intense vortex shock 5 sec., 12,000 × g centrifugal 5 min. The supernatant immediately transferred to a pre‐cooling EP tube, namely cytoplasmic protein. The sediment was suspended in 50 μl pre‐cooling NER, intense vortex 15 sec., incubated with 10 min. Repeat the procedure for four times, a total of 40 min. 12,000 × g centrifugal for 10 min., immediately transfer the supernatant to a new EP tube, namely nuclear protein. Nuclear protein and cytoplasmic protein were performed by Western blot analysis of p65 nuclear translocation.

### Statistical analysis

All data were expressed as the mean ± SD. Statistical analyses were performed using SPSS 13.0 (SPSS, Chicago, USA). Multiple group comparisons were performed using the one‐way analysis of variance test followed by the application of Bonferroni test for multiple comparisons. *P *<* *0.05 was considered significant.

## Results

### Selective CDK7 inhibitor BS‐181 suppressed both the IL‐1β, IL‐6, IL‐8 and RANKL transcript levels and the IL‐1β/IL‐6 secretion in LPS‐induced MH7A cells

**Figure 1:**
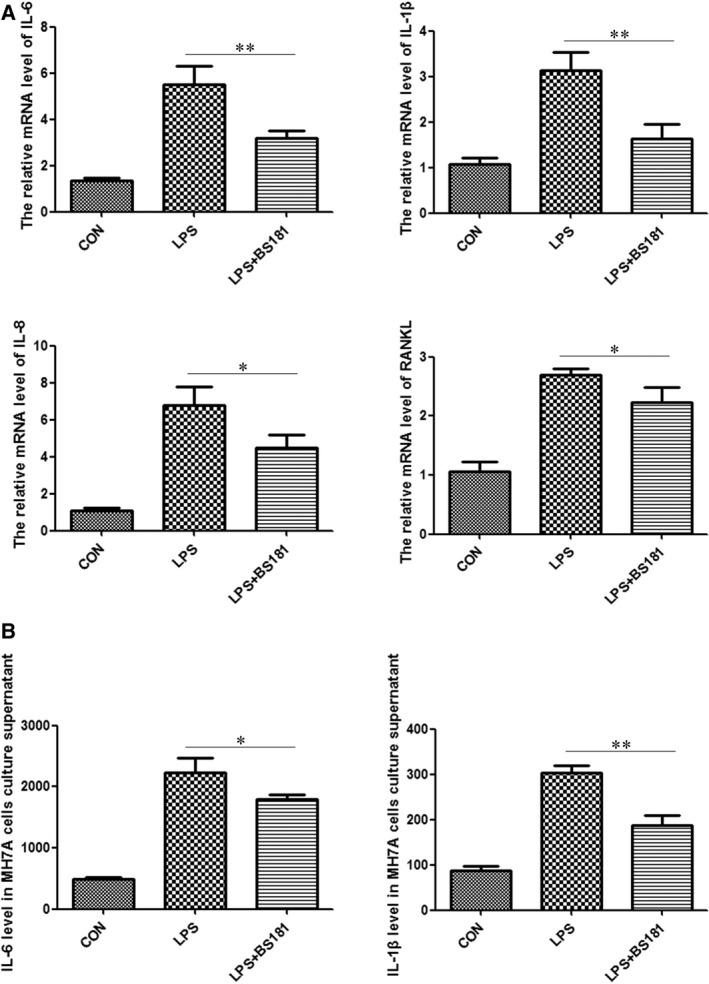
BS‐181 suppressed both IL‐1β, IL‐6, IL‐8 and RANKL transcript levels and the IL‐1β/IL‐6 secretion in LPS‐induced MH7A cells. (**A**) BS‐181 suppressed IL‐1β, IL‐6, IL‐8 and RANKL mRNA levels in LPS‐induced MH7A cells. The MH7A cells were stimulated by 1 μg/ml LPS and treated with 80 nM BS‐181 for 24 hrs. The IL‐1β, IL‐6, IL‐8 and RANKL mRNA levels were evaluated by quantitative real‐time PCR. Bar corresponds to mean ± SD. *P <* *0.05 and **P <* *0.01 as compared with control group. (**B**) BS‐181 suppressed IL‐1β/IL‐6 secretion in LPS‐induced MH7A cells. The MH7A cells were stimulated by 1 μg/ml LPS and treated with 80 nM BS‐181 for 48 hrs. Measurement of IL‐1β/IL‐6 secretion by ELISA. Bar corresponds to mean ± SD. *P <* *0.05 and **P <* *0.01 as compared with control group.

To clarify whether CDK7 inhibition directly affects RA inflammation *in vitro*, we took MH7A cells for research. MH7A cells were stimulated by 1 μg/ml LPS and treated with 80 nM BS‐181 for 24 hrs. IL‐1β, IL‐6, IL‐8 and RANKL mRNA levels were evaluated by quantitative real‐time PCR. As expected, the result showed that BS‐181 significantly down‐regulated the mRNA levels of IL‐1β, IL‐6, IL‐8 and RANKL, suggesting inhibition of CDK7 by BS‐181 effectively suppressed LPS‐induced MH7A inflammation (Fig. 1A). To further elucidate whether BS‐181 reduces secretion of IL‐1β and IL‐6 in LPS‐induced MH7A cells, we collected the supernatant of MH7A treated with 80 nM BS‐181 for 48 hrs and performed ELISA analysis. As consistent with the changes of IL‐1β and IL‐6 transcript levels, secretion of IL‐1β and IL‐6 was obviously inhibited as compared with control group (Fig. 1B).

### CDK7 knockdown decreased the IL‐1β, IL‐6, IL‐8 and RANKL transcript levels and IL‐1β/IL‐6 secretion in LPS‐induced MH7A cells

**Figure 2:**
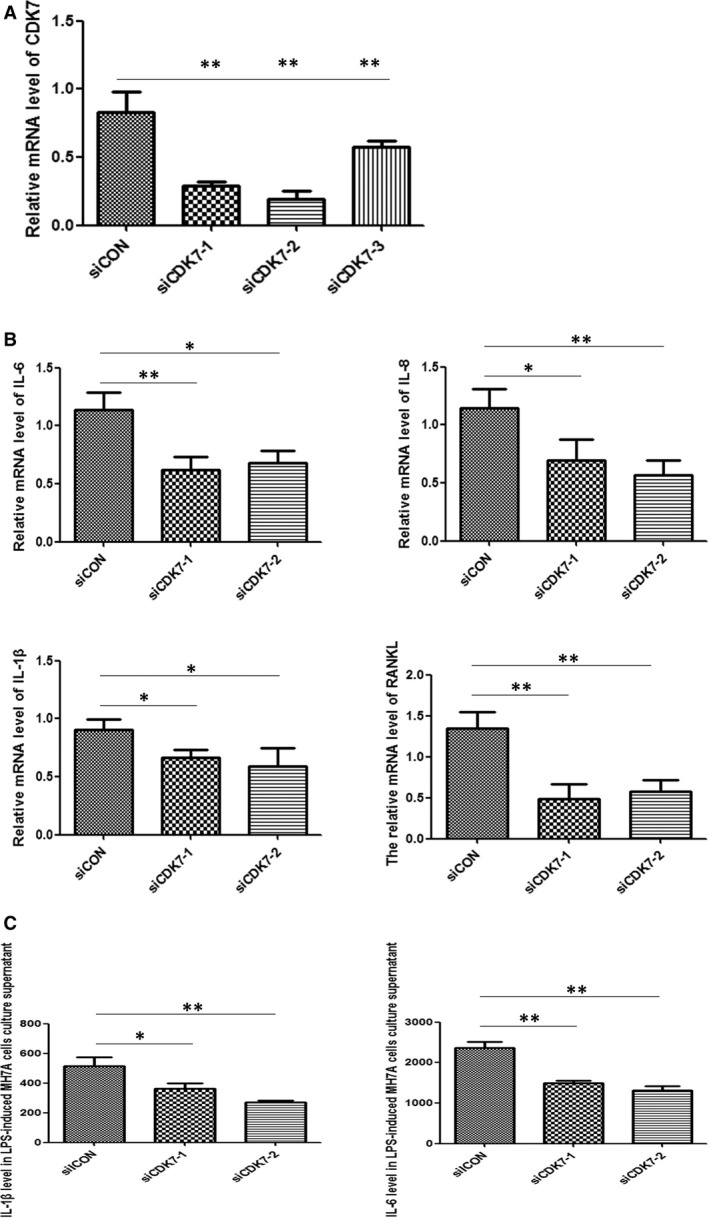
Knockdown of CDK7 decreased IL‐1β, IL‐6, IL‐8 and RANKL transcript levels and IL‐1β/IL‐6 secretion in LPS‐induced MH7A cells. (**A**) The efficiency of knockdown of CDK7 using siRNA. Three interfering fragments, siCDK7‐1, siCDK7‐2 and siCDK7‐3, were used for CDK7 silence. Bar correspond to mean ± SD. **P <* *0.01 as compared with control group. (**B**) IL‐1β, IL‐6, IL‐8 and RANKL transcript levels in LPS‐induced MH7A cells with siRNA‐CDK7. The MH7A cells were stimulated by 1 μg/ml LPS and treated with siCDK7‐1 and siCDK7‐2 for 24 hrs. The IL‐1β, IL‐6, IL‐8 and RANKL mRNA levels were evaluated by quantitative real‐time PCR. Bar corresponds to mean ± SD. *P <* *0.05 and **P <* *0.01 as compared with control group. (**C**) ELISA analysis for IL‐1β/IL‐6 secretion in LPS‐induced MH7A cells. MH7A cells stimulated with LPS in the presence or absence of siRNA‐CDK7 for 48 hrs. Bar corresponds to mean ± SD. *P <* *0.05 and **P <* *0.01 as compared with control group.

To further confirm the effect of CDK7 on MH7A cells, we used siRNA to knockdown CDK7. First, the siRNA‐1 and siRNA‐2 for CDK7 effectively silenced CDK7 mRNA level (Fig. 2A). The interference effect of siRNA‐3 for CDK7 decreased only about 40%. Therefore, siRNA‐1 and siRNA‐2 for CDK7 were used for following experiments. Second, by treatment with BS‐181, the result showed that CDK7 knockdown markedly decreased IL‐1β, IL‐6, IL‐8 and RANKL transcript levels in LPS‐induced MH7A cells (Fig. 2B). To evaluate whether CDK7 knockdown also reduces IL‐1β/IL‐6 secretion in LPS‐induced MH7A cells, we performed ELISA analysis to detect IL‐1β and IL‐6. As expected, knockdown of CDK7 significantly inhibited IL‐1β and IL‐6 levels in supernatant of LPS‐induced MH7A cells compared with control group (Fig. 2C). These data strongly suggested that CDK7 inhibition, by either treatment with BS‐181 or using siRNA, significantly suppressed the expression and secretion of IL‐1β and IL‐6.

### BS‐181 treatment and siRNA‐CDK7 blocked NF‐κB signalling pathway activation in LPS‐induced MH7A cells

**Figure 3:**
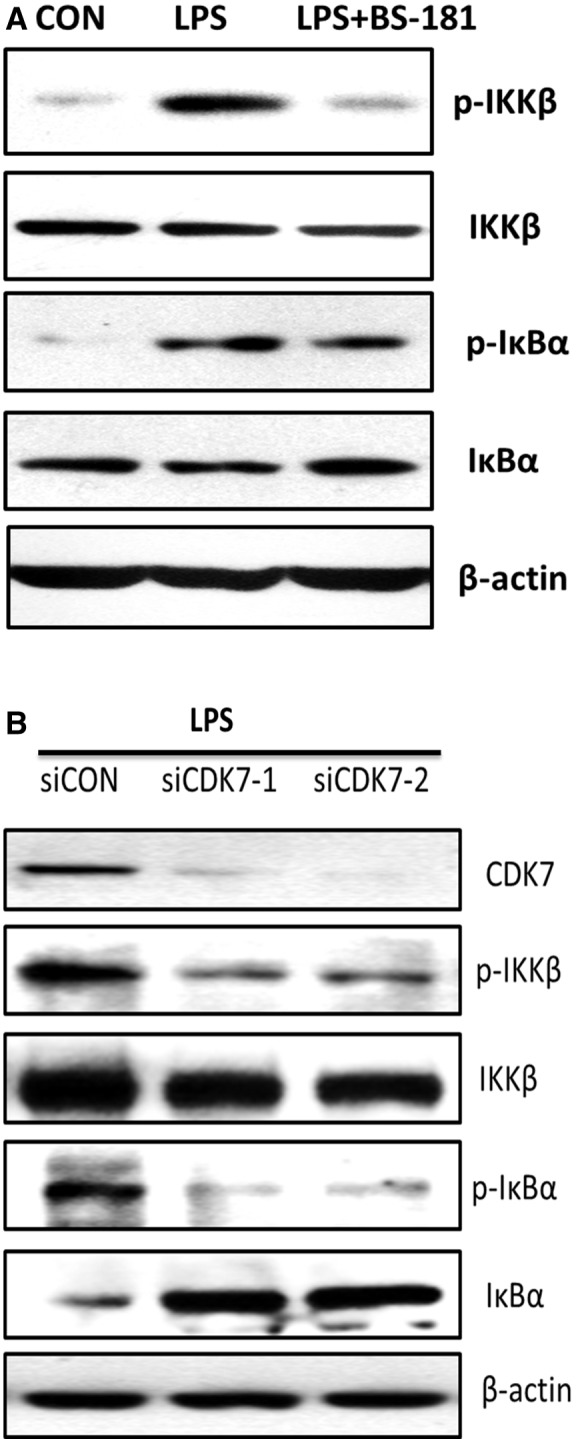
BS‐181 treatment and siRNA‐CDK7 blocked NF‐κB signalling pathway activation in LPS‐induced MH7A cells. (**A**) Western blot analysis of p‐IKKβ, IKK, p‐IκBα and IκBα protein levels in LPS‐induced MH7A cells treated with BS‐181 for 24 hrs. β‐actin was used as a loading control. (**B**) Western blot analysis of p‐IKKβ, IKK, p‐IκBα and IκBα protein levels in LPS‐induced MH7A cells treated with siRNA‐CDK7 for 24 hrs. β‐actin was used as a loading control.

The role of NF‐κB was critical in RA inflammation 24. To examine whether CDK7 inhibition leads to a decrease in IL‐1β/IL‐6 secretion due to blockage of NF‐κB signalling activation, we first tested NF‐κB signalling pathway‐related protein level in LPS‐induced MH7A cell treated with BS‐181. We observed that p‐IKKβ and p‐IκBα levels were decreased, and IκBα was up‐regulated in MH7A cells treated with BS‐181, suggesting that CDK7 inhibition by BS‐181 blocked NF‐κB signalling activation *in vitro* (Fig. 3A). To further identify the effect of CKD7 inhibition on blockage of NF‐κB signalling activation, we used siRNA for silencing CDK7. As consistent with the result of CKD7 inhibition by BS‐181, NF‐κB signalling was markedly blocked by siRNA‐CDK7 (Fig. 3B). These results suggest that CDK7 inhibition is responsible for the blockage of NF‐κB signalling activation *in vitro* and finally results in the down‐regulation of IL‐1β, IL‐6, IL‐8 and RANKL transcript levels and IL‐1β/IL‐6 secretion.

### Selective CDK7 inhibitor BS‐181 suppressed p65 nuclear translocation and phosphorylation of p65 in LPS‐induced MH7A cells

**Figure 4:**
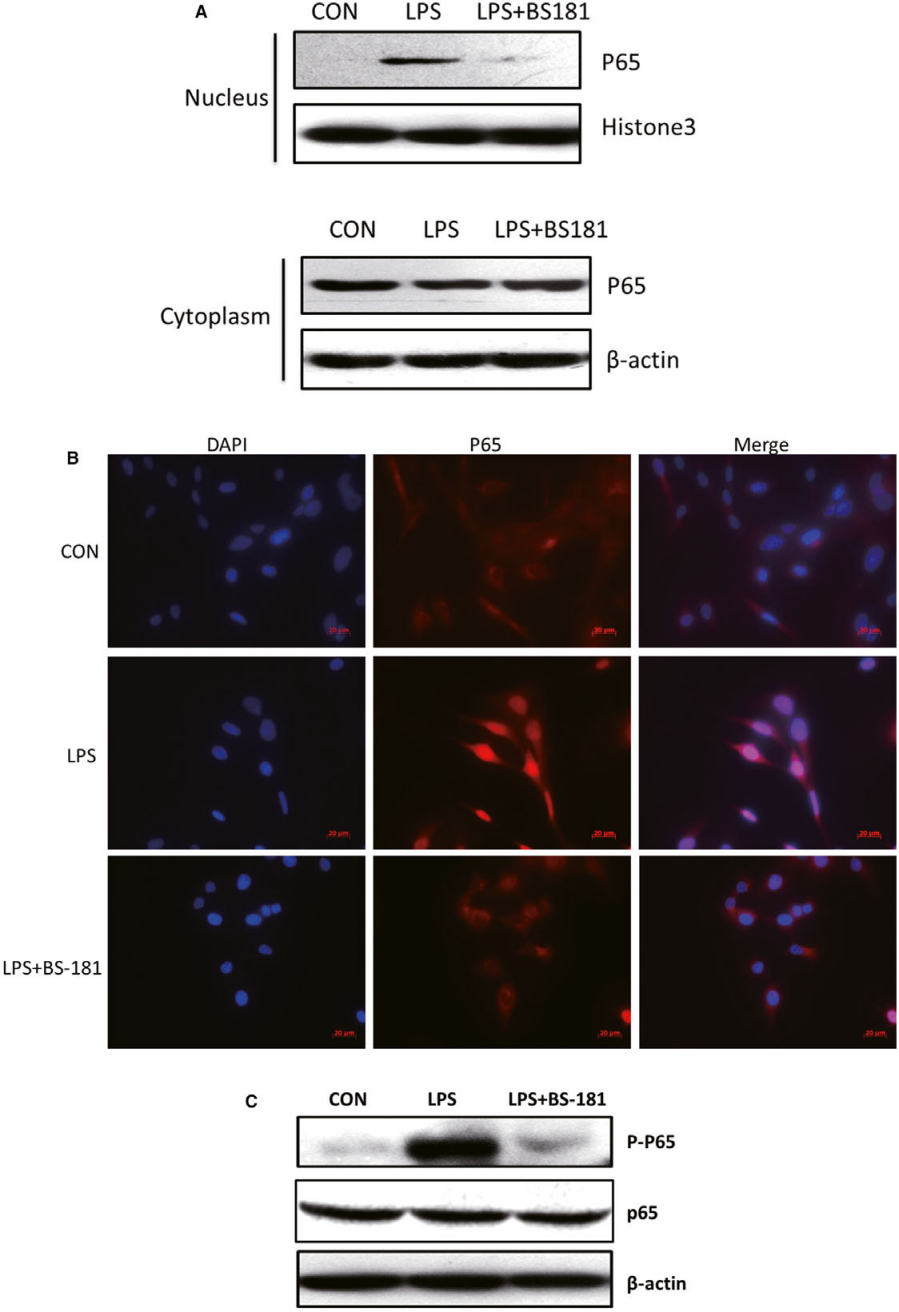
BS‐181 suppressed p65 nuclear translocation and phosphorylation of p65 in LPS‐induced MH7A cells. (**A**) Western blot analysis of p65 protein level in nuclear and cytoplasm of LPS‐induced MH7A cells treated with BS‐181 for 48 hrs. The p65 protein level was down‐regulated in nuclear and up‐regulated in cytoplasm. β‐actin and histone 3 were used as a loading control. (**B**) Fluorescent immunocytochemistry analysis of p65 protein level in nuclear and cytoplasm of LPS‐induced MH7A cells treated with BS‐181 for 48 hrs. The nuclear translocation of p65 was reduced in LPS‐induced MH7A cells treated with BS‐181. Scale bar: 20 μm. (**C**) Western blot analysis for p‐p65 in LPS‐induced MH7A cells treated with BS‐181 for 24 hrs. β‐actin was used as a loading control.

To evaluate the role of CDK7 inhibition by BS‐181 on p65 nuclear translocation, we extracted the nuclear protein and cytoplasmic protein for Western blot analysis. The result showed that BS‐181 significantly suppressed nuclear translocation of p65 and relatively up‐regulated p65 protein level in cytoplasm (Fig. 4A). Furthermore, the similar result was also obtained in immunofluorescent assay (Fig. 4B).

Besides phosphorylation and subsequent degradation of inhibitory molecular, protein kinases are also required for optimal NF‐κB activation by targeting functional domains of NF‐κB protein themselves 25. Therefore, we next examined the phosphorylation of p65 in LPS‐induced MH7A cells treated with BS‐181. We found that selective CDK7 inhibitor BS‐181 greatly decreased the phosphorylation of p65, suggesting that CDK7 inhibition strongly blocked NF‐κB signalling pathway activation (Fig. 4C).

### Selective CDK7 inhibitor BS‐181 decreased transcript levels of IL‐1β, IL‐6, IL‐8 and RANKL and IL‐1β/IL‐6 secretion in LPS‐induced FLS cells (fibroblast‐like synovial cells)

**Figure 5:**
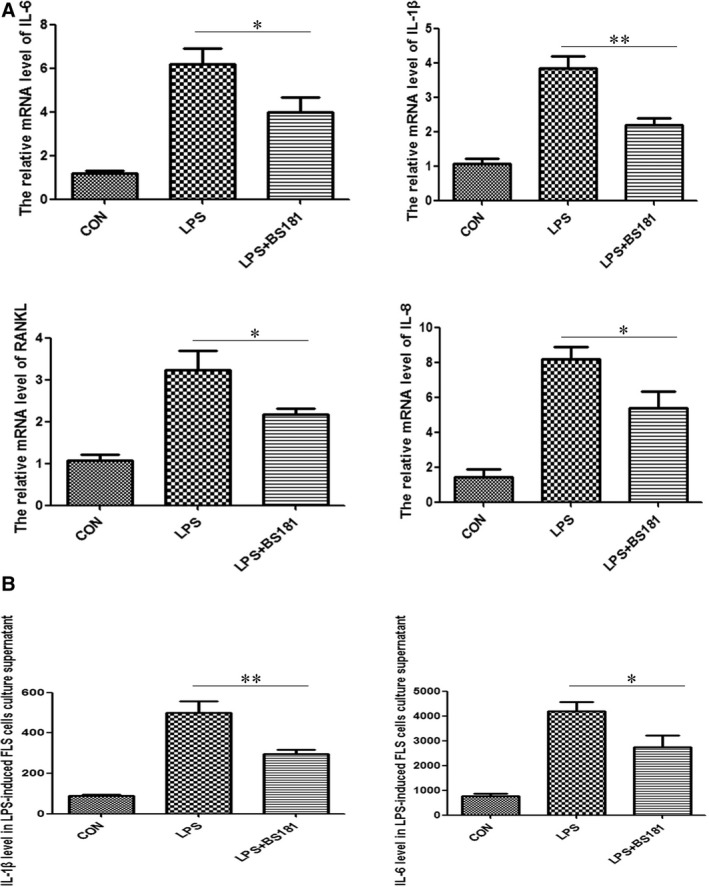
BS‐181 decreased transcript levels of IL‐1β, IL‐6, IL‐8 and RANKL, and IL‐1β/IL‐6 secretion in LPS‐induced FLS cells. (**A**) Real‐time PCR analysis for IL‐1β, IL‐6, IL‐8 and RANKL mRNA levels in LPS‐induced MH7A cells treated with BS‐181 for 24 hrs. The FLS cells were stimulated by 1 μg/ml LPS and treated with 80 nM BS‐181 for 24 hrs. The IL‐1β, IL‐6, IL‐8 and RANKL mRNA levels were evaluated by quantitative real‐time PCR. Bar corresponds to mean ± SD. *P <* *0.05 and **P <* *0.01 as compared with control group. (**B**) ELISA analysis for IL‐1β/IL‐6 secretion in LPS‐induced FLS cells. FLS cells simulated with LPS in the presence or absence of BS‐181 for 48 hrs. Bar corresponds to mean ± SD. *P <* *0.05 and **P <* *0.01 as compared with control group.

To further elucidate the universal effect of CDK7 on rheumatoid arthritis synovial fibroblast cells, we isolated and cultured fibroblast‐like synovial cells from CIA mice's joints. Similar to the result in MH7A cells, BS‐181 significantly down‐regulated transcript levels of IL‐1β, IL‐6, IL‐8 and RANKL in LPS‐induced FLS cells (Fig. 5A). To further evaluate whether BS‐181 reduces secretion of IL‐1β and IL‐6 in LPS‐induced FLS cells, we collected the supernatant of FLS cells treated with 80 nM BS‐181 for 48 hrs and performed ELISA analysis. As consistent with the changes of IL‐1β and IL‐6 mRNA levels, secretion of IL‐1β and IL‐6 was obviously inhibited as compared with control group (Fig. 5B), suggesting that CDK7 inhibition suppressed inflammation in RA synovial fibroblast cells.

## Discussion

Rheumatoid arthritis is a chronic, debilitating autoimmune disease characterized by synovial inflammation and destruction of joints. Persistent joint inflammation and progressive joint damage eventually result in disability and decreased quality of life 26, 27; many patients have to cope with pain, depression and fatigue 28. Biological therapies that target a specific inflammatory pathway could improve outcomes of RA patients. However, there is still a complete lack of response in a large number of patients to certain therapy 10. Thus, the needs for improved understanding of the disease at the basic molecular level and the search for new effective drugs are imperative. In recent years, the cyclin‐dependent kinase inhibitor (CDKi) drugs show promising anti‐inflammatory potentials in a number of human diseases 15, 29. In our previous study, we found that selective specific inhibition of CDK7 by BS‐181 significantly inhibited joint synovial inflammation of collagen‐induced arthritis (CIA) mice 22, but the underlying mechanism of CDK7 in RA is still unknown. In the present study, we discovered both IL‐1β, IL‐6, IL‐8 and RANKL transcript levels and IL‐1β/IL‐6 secretion were effectively suppressed in LPS‐induced MH7A cells and FLS cells by selective CDK7 inhibitor BS‐181 (Fig. 1). Meanwhile, the results were similar when we used siRNA to knockdown CDK7 (Fig. 2). Furthermore, our results also show that CDK7 inhibition significantly blocked NF‐κB activation and decreased p65 nuclear translocation (Figs 3 and 4), suggesting that CDK7 inhibition suppressed inflammation of RA through blockage of NF‐κB signalling pathway activation.

FLS cells have a dual role in RA, by responding appropriately to the inflammatory environment and through aggressive behaviour imprinted during their sojourn through the rheumatoid synovium 16. FLS cells normally assure the structural and dynamic integrity of diarthrodial joints by controlling the composition of the synovial fluid and the extracellular matrix (ECM) of the joint lining. However, FLS cells in patients with RA can be stimulated and increase the ability to secrete a variety of cytokines, chemokines and proangiogenic factors, such as IL‐1β, IL‐6, IL‐8, GM‐CSF, MMPs and TNFα 30. Moreover, these proinflammatory factors can be responsible for FLS invasiveness and bone destruction in RA 31, 32. Inhibition of RA FLS inflammatory factors secretion significantly improve signs and symptoms of RA 33, 34. Cyclin‐dependent kinases (CDKs) are a large group of serine/threonine protein kinases that have central roles in controlling the cell cycle and transcription 12, 13, 14. CDK7, firstly, acted as an essential component of the transcription factor TFIIH and involved in transcription initiation by phosphorylating the COOH‐terminal domain of the largest subunit of RNA pol II 14. Recently, studies showed that CDK7 inhibition could promote resolution of inflammation in bleomycin‐induced lung injury model *via* regulating neutrophil transcription 21. BS‐181, a pyrazolo [1,5‐α] pyrimidine‐derived compound, is a novel selective inhibitor of CDK7 by computer‐aided drug design 11. In the present study, we found that both IL‐1β, IL‐6 and IL‐8 transcript levels and IL‐1β/IL‐6 secretion were effectively suppressed in LPS‐induced MH7A cells and FLS by selective CDK7 inhibitor BS‐181 as well as CDK7 knockdown, which is coincident with that of CDK7 inhibition ameliorated experimental arthritis 22, suggesting that CDK7 inhibition strongly impeded RA inflammation. These findings extend the role of CDK7 in RA inflammation.

The NF‐κB pathway is a major regulator of proinflammatory cytokine production and is activated by IL‐1, TNF and TLR signalling. In FLS, signal through the NF‐κB pathway requires inhibitor of NF‐κB kinase subunit β (IKKβ) in the cytosol and is independent of IKKα 35. Activation of IKKβ leads to phosphorylation of proteins of the inhibitor of NF‐κB (IκB) family. IκB proteins form complexes with cytosolic subunits of NF‐κB, maintaining them in an inactive state—after phosphorylation, IκBs are degraded by the proteasome, leaving NF‐κB (p65) free to migrate into the nucleus and initiate gene transcription 36. In our study, we showed that CDK7 inhibition suppressed NF‐κB signalling pathway activation and p65 nuclear translocation in RA FLS (Figs 3 and 4), indicating that reduction of IL‐1β/IL‐6 secretion by CDK7 inhibition might be due to NF‐κB signalling pathway blockage. Furthermore, besides phosphorylation and subsequent degradation of inhibitory molecular, protein kinases are also required for optimal NF‐κB activation by targeting functional domains of NF‐κB protein themselves 25. Our data also displayed that CDK7 inhibitor BS‐181 greatly decreased the phosphorylation of p65 (Fig. 4C, further supporting CDK7 inhibition result in NF‐κB signalling pathway inactivation.

Our data showed that CDK7 inhibition prevented NF‐κB signalling pathway activation *via* inhibiting p‐IKKβ and restrained p65 nuclear translocation. But the mechanism by which CDK7 regulates p‐IKKβ remains unclear and deserves our further investigation. Furthermore, some studies show that CDK 4/6 inhibitor (CDKI), palbociclib, combined with cytokine blockade enhanced anti‐arthritic effects without increasing immune suppression in mice 15. It is worthy to further study whether the combination of other CDK7 inhibitor and DMARDs is effective in rheumatoid arthritis. Moreover, it is necessary to pay attention to the side effect profile of the combination of other CDK7 inhibitor and DMARD, which may include tolerable and limited gastrointestinal disturbance, skin rash, reversible transaminitis and hypokalaemia. Together, our results demonstrate that CDK7 inhibition can significantly suppress the inflammation of RA *via* blockage of NF‐κB signalling pathway activation and IL‐1β/IL‐6 secretion, indicating that CDK7 may be an attractive target for RA therapies in the future.

## Conclusion

Rheumatoid arthritis (RA) is a chronic inflammatory disease characterized by joint swelling, joint tenderness and destruction of synovial joints, leading to severe disability. Anti‐inflammatory drugs and DMARDs may improve RA process. However, in most patients the treatment effect is still not satisfactory. In our previous research, we found that inhibition of CDK7 by highly selective inhibitor BS‐181 significantly impeded the development of collagen‐induced arthritis (CIA) mice. However, the underlying mechanism of CDK7 in RA remains to be explored. In our current studies, we found that both IL‐1β, IL‐6, IL‐8 and RANKL transcript levels and IL‐1β/IL‐6 secretion were effectively suppressed by BS‐181 treatment as well as CDK7 knockdown. Furthermore, CDK7 inhibition prevented NF‐κB signalling pathway activation and restrained p65 nuclear translocation. Moreover, CDK7 selective inhibitor BS‐181 also blocked phosphorylation of p65 in MH7A cells. Together, our results demonstrate that CDK7 inhibition can significantly suppress the inflammation of RA *via* blockage of NF‐κB signalling pathway activation and IL‐1β/IL‐6 secretion, indicating that CDK7 may be an attractive target for RA therapies in the future.

## Competing interest

The authors confirm that there are no potential conflict of interests.
